# Spatial heterogeneity in climate change effects across Brazilian biomes

**DOI:** 10.1038/s41598-024-67244-x

**Published:** 2024-07-16

**Authors:** Adriano Braga, Márcio Laurini

**Affiliations:** Department of Economics, University of São Paulo, Av. dos Bandeirantes 3900, Ribeirão Preto, São Paulo 100190 Brazil

**Keywords:** Climate change, Spatial heterogeneity, Spatio-temporal models, Structural time series, Local climate change, Climate sciences, Atmospheric science, Environmental impact

## Abstract

We present a methodology designed to study the spatial heterogeneity of climate change. Our approach involves decomposing the observed changes in temperature patterns into multiple trend, cycle, and seasonal components within a spatio-temporal model. We apply this method to test the hypothesis of a global long-term temperature trend against multiple trends in distinct biomes. Applying this methodology, we delve into the examination of heterogeneity of climate change in Brazil—a country characterized by a spectrum of climate zones. The findings challenge the notion of a global trend, revealing the presence of distinct trends in warming effects, and more accelerated trends for the Amazon and Cerrado biomes, indicating a composition between global warming and deforestation in determining changes in permanent temperature patterns.

## Introduction

Climate change stands as one of the most pressing challenges of our era, wielding the potential to profoundly alter every ecosystem^1–8^ and significantly impact the health and prosperity of individuals worldwide^9^. Its effects reverberate across diverse biomes, disrupting delicate ecological balances and threatening countless species with extinction. Moreover, its far-reaching consequences extend beyond the natural world, as it poses grave risks to human health, livelihoods, and socio-economic stability.

Climate change profoundly influences ecological communities at a macroscopic level, triggering alterations across various functional dimensions. Arid and semi-arid regions witness heightened desertification, marked by escalated evaporation rates and diminished precipitation levels. This phenomenon exacerbates habitat depletion and elevates the susceptibility to dust storms^10^. Forest ecosystems undergo shifts in fire patterns, outbreaks of pests, and redistribution of species. Notably, wildfires increase in frequency and severity while invasive pests thrive in warmer climates^11–13^. Grasslands confront diminished productivity, augmented wildfire risks, and changes in plant diversity, adversely affecting indigenous grass species and associated fauna^14–17^. Aquatic habitats experience rising temperatures, ocean acidification, and sea level elevation, resulting in disruptions within food chains, declines in fish populations, and degradation of coastal ecosystems^18,19^.

Although the existence and effects of global climate change are well studied, especially the scientific consensus on the anthropogenic causes of climate change^20,21^, an important aspect about the impact of climate change is the spatial heterogeneity of these changes^22^, and how this heterogeneity can affect the human perception of these effects, even generating skepticism about the existence of climate changes^23^.

Our work contributes to the analysis of the spatial heterogeneity of climate change effects by proposing a spatio-temporal decomposition of the permanent and transient components of changes in climate patterns, allowing and testing for the existence of spatially localized components for these effects. As stated in^24^ (and references therein), one way to verify the existence of changes in climate patterns is through the estimation of trends and periodic components. In this work, we generalize this structure to estimate models with permanent (trend) and transitory (seasonal and cycle) components for each region (biome), comparing it with a model of unique global components, related to the hypothesis of spatial homogeneity in warming patterns linked to climate change. Our analysis is focused on testing whether the trend component, which captures non-reversible patterns of climate change^24,25^, is global or local. We analyze the hypothesis of a single global trend, implying spatial homogeneity of climate change, versus a hypothesis of multiple trends and spatial heterogeneity in climate change effects, through a Bayesian test using the Bayes Factor, and through information criteria (DIC and WAIC).

The trend components are modeled as a first-order random-walk process, a widely recognized approach in the time series and environmental analysis for the cumulative effect of all past shocks with lasting impacts^24,25^. Additional insights into the properties of this process, particularly in the context of temperature series trend modeling, can be found in^26^. The seasonality components are derived from mean effects per period, ensuring that these effects collectively balance to zero over the frequency of the series. To capture persistent dynamics with potential periodic patterns, we employ a formulation reminiscent of unobserved component models^27^. The cyclic component is characterized by a latent factor following a second-order autoregressive (AR) structure. The AR(2) model adeptly captures persistent but mean-reverting effects, particularly in cases involving complex roots within the polynomial of the associated lag operator generating periodic (cyclical) patterns. See Online Methods Section for a complete characterization of the model and the inference methods used.

As discussed in^24^, climate pattern inference usually faces some unconventional issues. The initial challenge is linked to the sources of information currently accessible, typically derived from numerous monitoring stations. While the abundance of climate data sources enhances accuracy in the inference processes, the statistical methods employed for trend and cycle extraction are often ill-suited to this characteristic since most of the statistical models are formulated for univariate or low-dimensional multivariate models.

The second challenge arises from the significance of the spatial distribution of climatic impacts, evident in the statistical examination of values recorded by the monitoring station network. Once more, conventional econometric methods utilized in structural (trend/seasonal/cycle) time series decompositions, including most Kalman filter-based approaches, struggle to adequately integrate spatial effects^24,28^.

Additionally, there is a practical challenge tied to the operational patterns of weather monitoring stations. Many of these stations operate automatically and are often situated in hard-to-reach areas. Operational issues may render these stations inoperative, resulting in data collection failures. Moreover, there is the continual addition and replacement of stations in new regions. From a statistical perspective, this translates to a substantial portion of missing data in the time series collected by these stations.

Extending^24^, our method combines elements of structural time series decomposition (e.g.,^29^) and the spatio-temporal models with continuous spatial random effects using the representation introduced by^30^, allowing us to deal simultaneously with all three characteristics issues of climate patterns inferences discussed above. Here, the difference resides in allowing for multiples trends and then testing its suitability for identifying climate change heterogeneity.

The spatio-temporal decomposition methodology proposed in^24^ was applied and generalized to other relevant contexts of spatio-temporal modeling of climate and environmental processes. Versions of this model for spatio-temporal point processes were applied to model the intensity of occurrence of tornadoes due to the effects of climate change^31^, in the analysis of impacts of regulatory changes on the burning of sugar cane on the occurrence of fires in the state of São Paulo-Brazil^32^, in modeling the intensity of fires in Australia^33^ and the Brazilian Amazon^34^, and in modeling changes in climate patterns of rainfall, temperature, record temperatures and rainfall and other climate effects in^35^, being a consolidated methodology for spatio-temporal analysis of climate and environmental effects.

We analyze the spatial heterogeneity in temperature growth patterns between the six distinct biomes in Brazil–Caatinga, Cerrado, Pantanal, Pampa, Amazônia and Atlantic Forest for the period 1961–2023. The Caatinga is a dry forest biome that covers parts of northeastern Brazil. It is characterized by its thorny vegetation, drought-resistant plants, and seasonal rainfall patterns^36^. The Cerrado is a vast tropical savanna biome that covers a large portion of central Brazil. It is characterized by a mix of grasslands, shrublands, and forests, and is considered one of the world’s most biodiverse savannas^37^. The Pantanal is the world’s largest tropical wetland area, located primarily in western Brazil. It is renowned for its rich biodiversity, including numerous species of birds, mammals, and aquatic animals, and serves as an important habitat for migratory birds^38^. The Pampas is a vast biome that extends across southern Brazil, as well as parts of Uruguay and Argentina. It is primarily composed of grasslands and is known for its fertile soil^39^. The Amazon is the largest tropical rainforest in the world, covering a significant portion of Brazil’s northern region. It is known for its unparalleled biodiversity, with millions of plant and animal species, as well as its vital role in regulating the Earth’s climate^40^. The Atlantic Forest is a biome that once stretched along Brazil’s Atlantic coast but has been significantly reduced due to deforestation. It is known for its high levels of biodiversity and unique ecosystems, including dense forests, mangroves, and coastal dunes^41^.

The results obtained in our analysis indicate a rejection of the hypothesis of spatial homogeneity in the warming trend, revealing significant variation in warming patterns between 1961 and 2023. Particularly noteworthy is the substantial increase in temperature trends observed in the Amazon Biome, with a rise of 1.12 °C, and in the Cerrado, showing a growth of 0.85 °C. In contrast, the Pantanal experienced a modest growth of 0.17 °C, while the Pampa saw a slightly higher increase of 0.37 °C. These findings potentially suggest a compounding of effects between global warming and accelerated deforestation in the Brazilian biomes.

The relationship between deforestation and climate change, with emphasis on global and regional warming patterns, occurs through several channels. The first channel is through the role of carbon sequestration, since forests act as carbon sinks, absorbing carbon dioxide (CO_2_) from the atmosphere during the process of photosynthesis^42,43^. This helps to mitigate the greenhouse effect, which is a major driver of global warming. When forests are cut down or burned, the stored carbon is released back into the atmosphere as CO_2_, a potent greenhouse gas^44^. This release of carbon increases atmospheric CO_2_ concentrations, contributing significantly to global warming. In this aspect there is a dependence between deforestation and fire occurrence, since the use of fire is a common way of carrying out deforestation, as discussed for the Amazon biome in^34^, for the Pantanal in^45^ and for the Cerrado in^46^. In addition to affecting the exchange of carbon dioxide (CO_2_) and moisture with the atmosphere and surface albedo, vegetation emits biogenic volatile organic compounds (BVOCs) that alter the formation of short-lived climate forcers (SLCFs), which include aerosol, ozone and methane^47^.

Deforestation can trigger feedback loops that amplify global warming. For example, as temperatures rise due to increased CO_2_ levels, forested areas can become more susceptible to fires^48,49^. These fires release even more CO_2_, creating a vicious cycle of warming and forest loss. Additionally, deforestation in tropical regions can reduce the rainfall needed to sustain remaining forests, leading to further deforestation and CO_2_ emissions. Deforestation can also alter local and regional climates by disrupting the water cycle^50,51^. Trees play a crucial role in maintaining the hydrological cycle by absorbing water from the soil and releasing it into the atmosphere through transpiration. This process contributes to cloud formation and precipitation. Without forests, regions can experience reduced rainfall, leading to drier climates and more frequent droughts, which further exacerbate climatic changes. Evidence of changes in the water cycle in the Amazon are studied in^52–54^ and for the Cerrado biome in^46,55–57^.

A further channel through which deforestation affects climatic conditions is by disturbing rainfall patterns. Mu and Jones^58^ showed that rainfall trends in Brazilian Legal Amazon are heterogeneous, lacking a consistent pattern across the entire region. Nevertheless, they found coherent connections between declining dry-season rainfall and old-age deforestation areas. This means that, in these areas, dry-season are getting drier. Their results also indicate that deforestation effects have also temporal variability, with deforested areas up to a decade increasing dry-season rainfall and older deforested regions experiencing reduced rainfall during the dry season. Leite-Filho^52^ pointed that deforestation impact on rainfall also depends on its extension, showing that rainfall diminishes after deforestation surpasses certain threshold, but this boundary is lower at larger scales. The results are also consistent with^59^. Werth and Avissar^60^ simulated deforestation scenarios and pointed that deforestation effect in the Amazon is quite relevant, potentially leading to decreases in rainfall, evapotranspiration, and cloudiness. They also observed impact in various other regions worldwide, many of which exhibit a reduction in rainy season precipitation.

Based on simulations from the Brazilian Earth System Model version 2.5 (BESM 2.5) under the RCP8.5 scenario, Gomes^61^ finds that escalating global greenhouse gases and land cover change due to deforestation have significant impacts on the water budget in the Amazon basin. These factors lead to a substantial increase in temperature across the basin, up to 5 °C, driven by changes in energy and water budgets. Under high greenhouse gas emissions scenarios, there are reductions in moisture convergence, precipitation, and evapotranspiration, primarily due to a positive feedback loop. Conversely, in scenarios of future deforestation, precipitation reductions are even more pronounced, driven by a negative feedback mechanism. Hofmann et al.^62^ revealed a significant reduction in both rainfall and the frequency of rainy days in the northern and central regions of the Cerrado for all periods, except at the beginning of the dry season. The most pronounced negative trends were observed during the dry season and the early wet season, with reductions of up to 50% in total rainfall and the number of rainy days. Hofmann et al.^62^ attributed to the intensification of the South Atlantic Subtropical Anticyclone, which has altered atmospheric circulation and increased regional subsidence. Additionally, reduced regional evapotranspiration during the dry season and early wet season likely contributed to the observed rainfall reduction.

The study by^63^ indicates that the process of savannization and the phenomenon of global warming independently extend the duration of the dry season and decrease annual rainfall across extensive areas of South America. When considering the combined impacts of land use changes and global warming,^63^ found a significant reduction in mean annual rainfall by 44% and an increase in dry season length by 69%, on average across the Amazon basin, compared to the control scenario. The alteration of inland moisture transport patterns due to savannization emerges as the primary factor driving the observed reduction in rainfall and the lengthening of the dry season over the Amazon and Central-West regions. A related result is presented in^64^, that shows that forests exhibit greater susceptibility to climate change compared to savannas or grasslands. Forested areas demonstrated lower resilience to climatic stress and a heightened likelihood of encountering non-analogous climatic conditions. In such a scenario, forest ecosystems would face greater challenges in adapting compared to savannas or grasslands, primarily due to their narrower climate niche.

Similar results were found for the Cerrado biome. Hofmann et al.^62^ indicates the impact of changes in evapotranspiration patterns on the Cerrado rainfall regime, and^65^ provides evidence that the expansion of cropland in the Cerrado has led to deforestation, which increasingly affects rainfall patterns. Changes in land use and impacts of climate change have significantly impacted river flows in the Cerrado, with large-scale deforestation aimed at the production of irrigated agricultural commodities having a more significant impact on river flows than climate changes, as shown by^57^. Similar results for the biome were found in^66,67^.

The effects of land use change and deforestation on the rainfall pattern in the Caatinga are analyzed in^68^, and the anthropogenic effects on vegetation patterns in this biome are examined by^69^. The negative impact of deforestation on rainfall is also documented for the Atlantic Forest biome^70^, and for the Pantanal^71,72^.

An additional important consideration lies in understanding the connection between tree cover and climate patterns, as examined in^73^. This study investigates how three primary forms of vegetation cover–forests, savannas, and treeless landscapes–influence rainfall patterns and their respective resilience to climate change. Notably, the findings in^73^ highlight the anticipated diminished resilience of forested areas in the central eastern and southeastern Amazon basin, which aligns with the increased human activity observed within the deforestation arc. These specific regions are identified as particularly vulnerable to heightened drought risks in future climate projections.

The Albedo effect is another local factor linking deforestation and climate change^74^. Forests typically have a lower albedo compared to other land covers, meaning they absorb more sunlight. However, when forests are cleared and replaced with agricultural land or urban areas, the albedo of the land increases, causing it to reflect more sunlight back into space^75^. While this may initially appear to have a cooling effect on the Earth’s surface, it is outweighed by the loss of forests’ ability to sequester carbon dioxide, resulting in an overall warming effect on global temperatures.

The results presented by^76^ suggest varying vulnerabilities among South American biomes, with those located in the tropical belt likely facing higher temperatures compared to temperate biomes. Additionally, forested biomes are projected to experience greater moisture loss than those with open or desert vegetation. Furthermore, higher vegetation density correlates with a more substantial reduction in humidity, indicating that forest biomes may encounter the most significant decrease in precipitation relative to open vegetation biomes. These predictions align with previous studies by^77^ and^78^, which suggest that warmer and drier conditions could facilitate the expansion of open area biomes at the expense of forested biomes. Moreover, these effects may be exacerbated when coupled with significant land-use changes, as highlighted by^79^.

The work of^80^ employ the theory of critical transitions to analyze and project significant changes in Brazilian biomes. In the Amazon, climate change is leading to increased tree mortality, reduced forest biomass, and more frequent fire episodes, resulting in biodiversity loss and diminished ecosystem services. Projections for the Caatinga suggest a transition to a more desert-like state, characterized by high temperatures and critical rainfall levels, indicating a tendency towards aridization and expansion of desert areas due to environmental degradation. In the Cerrado, projections indicate expansion into areas previously occupied by the Amazon biome and the Atlantic Forest, particularly under warming scenarios of 1.5 °C and 2 °C. The Atlantic Forest experiences a decline in biome occupation and significant resilience loss, with low resilience dominating most areas, while intermediate and high resilience are concentrated along the coast. The Pampa biome loses its high resilience at all warming levels and contracts to a small fraction in the far south, expanding into former Cerrado areas with low resilience. Modeling indicates an increasing vulnerability to climate change in the Pampa biome due to projected temperature increases and extreme rainfall events, impacting resilience and adaptive capacity.

The findings from our analyses align with previous studies investigating the effects of climate change on Brazilian biomes. Specifically, our results substantiate the combined influence of global warming and local deforestation on the observed temperature rises within the Amazon biome^81–84^, along with their consequential impacts on rainfall variability and wildfires in this region^34,85^. Moreover, our results also underscore the significance of deforestation and land use patterns in shaping temperature trends across various other biomes including the Cerrado^86^, Atlantic Forest^87^, Caatinga^88^, Pampa^89^, and Pantanal^72,90^.

Our methodology permits us to gather evidence and rigorously test hypotheses concerning climate change patterns in climate series. This statistical rigor is vital for both quantifying and qualifying various facets of climate change. The outcomes derived from our approach can help to monitor and discern between permanent and transient effects within climate series. This delineation is crucial for informing the development of policies aimed at both mitigating and reversing the impacts of climate change, grounded firmly in robust statistical evidence. This monitoring of permanent and transitory patterns is essential to analyze the impacts of climate change on biodiversity and ecosystems^91,92^, food security^93^, health^94^ and economics^95^.

## Methodology and data

### Methodology

To analyze the spatial heterogeneity in warming we generalize the model proposed by^24^, using a structure of multiple trend, seasonality and cycle components combined with the continuous spatial random effects introduced by^30^. We allow each biome to have specific latent components, enabling spatial heterogeneity in climate change patterns. To estimate the latent components and the related parameters we apply a Bayesian inference procedure using integrated nested Laplace approximations (INLA). For a detailed exploration of the computational properties of INLA see^24,96,97^.

Within this continuous spatial random effects structure, estimates for missing data can be derived by combining the components of trend, seasonality, and cycle with the prediction obtained for the spatial effect at the station’s coordinates through continuous spatial projection. Consequently, the model does not necessitate additional procedures for handling missing data or alternative spatial interpolation structures^24^.

The model can be summarized by the structure:

1 $$\begin{array}{*{20}l} {y\left( {s,t} \right) = \alpha _{b} + \mu _{{t,b}} + s_{{t,b}} + c_{{t,b}} + z\left( {s,t} \right)\beta + \xi \left( {s,t} \right) + \varepsilon \left( {s,t} \right)} \hfill \\ {\mu _{{(t,b)}} = \mu _{{(t - 1,b)}} + \eta _{{\mu b}} } \hfill \\ {s_{{(t,b)}} = s_{{(t - 1,b)}} + s_{{(t - 2,b)}} + ...\,s_{{(t - m - 1,b)}} + \eta _{{sb}} } \hfill \\ {c_{{(t,b)}} = \phi _{1} c_{{(t - 1,b)}} + \phi _{2} c_{{(t - 2,b)}} + \eta _{{cb}} } \hfill \\ {\xi \left( {s,t} \right) = \omega \left( {s,t} \right)} \hfill \\ {Cov\left( {\omega \left( {s,t} \right)} \right) = {\mathcal{C}}\left( h \right)} \hfill \\ \end{array}$$

where $y\left( s,t\right)$ represents the value of observation *y* at location *s* and in period *t*, $\mu _{(t,b)}$, $s_{(t,b)}$ and $c_{(t,b)}$ are the components of trend, seasonality and cycle for each biome b, with independent Gaussian innovation components $\eta _{\mu b}$, $\eta _{s b}$ and $\eta _{c b}$; $z\left( s,t\right)$ is a set of covariates observed in the location *s* and period *t*, $\epsilon \left( s,t\right)$ is a spatial white noise representing the non-structured spatial errors, and $\xi \left( s,t\right)$ represents the spatial random effects following a spatially continuous Matérn covariance function ${\mathcal {C}}\left( h\right)$ and *h* a Euclidian distance. Details on the covariance function are showed in Appendix 1.

The structural decomposition specification for the time series common components is analogous to the basic structural model of^29^. The trend series $\mu _{(t,b)}$ is modeled as a first-order random-walk process, a common specification in the literature to represent permanent trends. The random-walk process can be seen as the accumulation of all shocks that occurred in the past with non-transitory effects. See also^26^ for other properties of this process on the modelling of trends for temperature series. The seasonality $s_{(t,b)}$ is based on mean effects by period, with the constraint that these effects must sum to zero on the frequency of the series. To capture a persistent but mean-reverting dynamics with a potential periodic pattern, we adopt a formulation akin to unobserved component models^27^ for $c_{(t,b)}$. The cyclic component is depicted by a latent factor exhibiting a second-order autoregressive (AR) structure. The AR(2) can capture cyclic patterns in the presence of complex roots for the associated lag operator’s polynomial. In the estimation we use a formulation using partial autocorrelations (PACF) to represent the cycle parameters.

The decomposition structure used in this work is based on some important assumptions. We assume that the spatial variability of temperature is partially explained by latitude effects, and that the remaining spatial effects are captured by the spatial random effect. In this aspect we assume that the spatial random effects are constant over time, and thus the patterns of spatial variability do not change, and all the dynamics of change are given by the dynamics of the latent components of trend, seasonality and cycle. We tested this assumption by estimating a version of the model with time-varying spatial random effects, using the proposed structure in^33^ and^34^, but the results obtained indicate evidence in favor of the formulation with time-constant spatial effects.

To estimate the model, we use a Bayesian estimation using the integrated nested Laplace Approximation (INLA) proposed by^96^, using the prior structure proposed by^24^. Details about the estimation method can be seen in^24,96,97^.

To test the null hypothesis of a homogeneous single trend in warming for all Brazilian Biomes against the alternative hypothesis of multiple trends we formulate a Bayesian test using the Bayes factor, and compare unrestricted (multiple trends) and restrict (single Trend) models using the DIC^98^ and WAIC^99^ Bayesian information criteria. The Bayes Factor ($BF$) is a measure used in Bayesian statistics to compare the likelihood of two competing hypotheses. It is the ratio of the marginal likelihoods of the data under each hypothesis^100^. We apply the test by calculating the log Bayes Factor by subtracting the logarithm of the likelihood of Hypothesis 2 from the logarithm of the likelihood of Hypothesis 1, with Hypothesis 1 corresponding to multiple trends (spatial heterogeneity in the trend warming) and Hypothesis 2 is the assumption of a unique global trend (spatial homogeneity in trend warming).

### Brazilian biomes and data

#### The Brazilian biomes

The Caatinga biome is characterized by distinct climatic features that shape its ecosystem^101–103^. The Caatinga territory comprises three distinct Köppen climatic classifications^104^—BSh: Hot semi-arid climate BWh: Hot desert climate, and Aw: Tropical savanna climate. The Caatinga experiences high temperatures throughout the year, with averages ranging between 23 °C and 27 °C. During the hottest months, temperatures can exceed 40 °C. There is a notable temperature variation between day and night, but seasonal temperature changes are less pronounced. Annual precipitation is low, ranging from 300 mm to 800 mm. This makes the region one of the driest in Brazil. Rainfall is highly variable and unpredictable. It is often concentrated in a short rainy season lasting 3 to 5 months, with the remainder of the year being extremely dry. Prolonged droughts are common and can last for several years, significantly impacting the ecosystem and local agriculture. The dry season is prolonged and severe, typically lasting 7 to 9 months. Due to the high temperatures and low humidity, evaporation rates are high. This contributes to water scarcity even during the rainy season. Relative humidity is generally low, particularly during the dry season, which exacerbates the arid conditions. The soils in the Caatinga are typically shallow, rocky, and nutrient poor. They have low water retention capacity, which further limits the availability of water for plants. The vegetation is adapted to arid conditions, featuring xerophytic (drought-resistant) plants such as cacti, thorny shrubs, and small, hardy trees. Many plants have deep root systems, thick bark, and small leaves to minimize water loss.

The Cerrado biome in Brazil, often referred to as the world’s most biodiverse savanna, exhibits distinctive climatic features that significantly influence its unique ecosystem^105,106^. The climatic heterogeneity of this biome can be observed through the different Koppen classifications that encompass this biome. Cfb: Oceanic climate with warm summers, Cfa: Humid subtropical climate with hot summers, Cwb: Subtropical highland climate with dry winters, BSh: Hot semi-arid climate, as described above and Cwa: Humid subtropical climate with dry winters. The average annual temperature in the Cerrado ranges from 20 to 26 °C. The hottest months are typically September and October, just before the onset of the rainy season, while June and July are the coolest months. The Cerrado experiences a pronounced wet-dry seasonal cycle. The rainy season occurs from October to April, accounting for about 90% of the annual precipitation, which ranges between 800 and 2000 mm. The dry season, from May to September, sees very little rainfall, often leading to drought conditions. Due to the proximity to the Amazon rainforest, moisture is often transported by wind to the Cerrado, making it the most humid savanna globally. This humidity is crucial during the wet season as it supports the dense growth of vegetation and the replenishment of water resources. Fire is a natural and essential part of the Cerrado’s ecology^107^. The dry season, with its lower humidity and accumulation of dry biomass, creates conditions conducive to wildfires, which help maintain the savanna’s structure by clearing old vegetation and promoting new growth. Many plant species in the Cerrado are adapted to withstand and even thrive after fire events.

The Pantanal biome in Brazil, recognized as the world’s largest tropical wetland, is characterized by a distinctive climatic pattern that influences its unique ecosystem^71,108^. The Koppen classification for the Pantanal is Aw: Tropical savanna climate, a hot climate with distinct wet and dry seasons. The Pantanal experiences an annual average temperature of about 26.7 °C. The warmest months are from October to March, with average highs around 33–34 °C. Conversely, the coolest months are June and July, where temperatures can drop to an average low of 18–19 °C In the northern parts of the Pantanal, temperatures can soar to 40 °C during the hottest periods. In the southern regions, winter nights can occasionally see temperatures plummeting to near freezing, influenced by cold air masses from Argentina. The Wet Season extends from October to March, and this period is marked by heavy rainfall and flooding. The average annual precipitation ranges from 1,000 to 1,300 millimeters, with peak rainfall occurring between November and March. Floodwaters inundate the landscape, transforming it into a vast aquatic habitat, which supports the area’s rich biodiversity. During the dry season, water levels recede, revealing extensive grasslands and savannas. This period experiences minimal rainfall, with June and July being the driest months. The dry season is also marked by cooler temperatures, especially during the nights.

The Pampa biome, also known as the Pampas, is characterized by a temperate climate with well-defined seasons, which significantly influences its unique grassland ecosystem^39^. The Köppen classification is Cfa: Humid subtropical climate with hot summers. The Pampa experiences a temperate climate with distinct seasonal variations. The average annual temperature ranges from 16 to 18 °C. The summers (December to February) are warm with average temperatures around 22 to 24 °C, although maximum temperatures can occasionally exceed 30 °C, and winters (June to August) are mild to cool, with average temperatures ranging from 10 to 12 °C. Minimum temperatures can sometimes drop to near freezing, especially during cold fronts. While there is no extreme wet season, spring (September to November) and autumn (March to May) typically receive slightly higher rainfall, which supports the growth of grasses and other vegetation. Spring and autumn are characterized by moderate temperatures and significant rainfall, fostering lush grasslands. There can be short dry spells, especially in the summer months, but these are not as pronounced as in other Brazilian biomes like the Caatinga or Cerrado. The Pampa biome experiences four well-defined seasons. This seasonal variability contributes to a diverse range of flora and fauna adapted to these changing conditions.

The Amazonia biome, encompassing the Amazon rainforest, is characterized by a hot and humid tropical climate with consistent high temperatures and significant rainfall throughout the year^109,110^. The Koppen classifications for the Amazon Biome are Aw: Tropical savanna climate, Am: Monsoon climate, and Af: Tropical rainforest climate. The region’s average temperature ranges between 25 °C and 28 °C. Rainfall is abundant, typically ranging from 2000 to 3000 mm annually, with some areas receiving even more. This precipitation is relatively evenly distributed, although there are distinct wet and dry seasons. The wet season usually occurs from December to May, while the dry season spans from June to November, though even during the dry season, rainfall is common. The Amazon basin’s humidity levels are high, often reaching 80% to 90%, which supports the dense, diverse vegetation of the rainforest. The biome’s climate contributes to its vast biodiversity, making it one of the richest ecosystems on Earth, with thousands of plant species and a multitude of animal species, many of which are endemic to the region.

The Atlantic Forest in Brazil is a diverse and complex biome with distinct climatic features^111,112^. Covering regions of eastern Brazil, it extends into Paraguay and Argentina. The extension of this biome is reflected in the heterogeneity of climate classifications: Cfb, Cfa, Cwb, BSh, Cwa, Aw, Am, and Af. This biome features a warm and humid climate. The average temperatures range between 20 °C and 25 °C, with variations depending on altitude and proximity to the coast. Coastal areas tend to be warmer and more humid, while higher altitudes, such as the Serra do Mar mountain range, experience cooler temperatures. The Atlantic Forest experiences high levels of rainfall, typically ranging from 1200 to 2800 millimeters annually. The precipitation is relatively evenly distributed throughout the year, though certain areas, such as the northern Zona da Mata, receive more rainfall from May to August due to the trade winds. Although the Atlantic Forest does not have extreme seasonal variations, it does exhibit some seasonality with a more pronounced wet season in the southern winter and a slightly drier season in the summer. However, the biome remains relatively humid year-round. The diverse topography of the Atlantic Forest, which includes mountains, valleys, and coastal regions, creates various microclimates. These range from the humid tropical rainforests near the coast to the cooler montane forests at higher elevations. Due to the dense forest cover and high levels of precipitation, the Atlantic Forest maintains high humidity levels throughout the year, contributing to its lush and diverse vegetation.

#### Data

In this study, we utilize weekly meteorological information sourced from the National Institute of Meteorology (INMET) via the BDMEP system for the period 1961–2023. This system supplies meteorological data from conventional and automatic meteorological stations within the INMET station network. The BDMEP system offers access to daily and monthly data from 1961 onwards. The analyzed data comes from 1069 conventional and automatic stations, and the total sample consists of 999,099 observations. The sample is unbalanced since the operation of the stations can occur at different periods.

**Figure 1:**
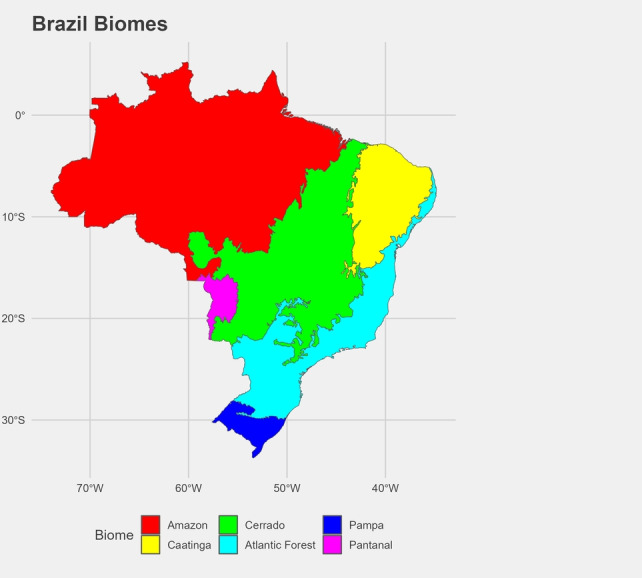
Brazil biomes. The figure shows the spatial delimitation of the six Brazilian biomes analyzed.

We present the estimation results for all six biomes existing in Brazil for the series of weekly average temperature. Figure 1 shows a map of the Brazilian Biomes. Following^24^ we define the trend components with a weekly frequency, and the seasonality and cycle components with a monthly frequency. This mixed data sampling structure allows estimating the trend components with the greatest possible precision, and the monthly frequency allows a direct interpretation for the seasonality and cycle components.

The entire territory of Brazil exhibits significant inter annual variability, characterized by extremes of both dry and rainy years, accompanied by pronounced inter seasonal fluctuations. Analyzing historical data for the entire country, the average temperatures typically span from 20 to 28 °C. However, specific regions such as the Chapada Diamantina and Borborema Plateau, situated at higher elevations, experience average temperatures consistently below 20 °C.

Our analysis only uses data from meteorological stations, while it would be possible to use other data sources such as remote sensing images or reanalysis datasets, combining observations from satellites and conventional ground-based stations, as for example in the analysis of climate patterns in the Cerrado used in^113^. Although it is possible to use these data sources in our analysis, these data structures initially present some difficulties for the estimation structure used in our article.

Reanalysis methods use additional data combination structures that can affect the estimation process, in particular spatially smoothing the observed data through the use of the theoretical climate model in spatial interpolation in the construction of the data grid. Our analysis allows us to directly estimate the precision involved in estimating the parameters and latent components of the model, quantifying the limitations placed by the observed data. The use of reanalysis data can lead to a biased estimate of the precision/variability of the estimated components in time and space, and to avoid this limitation we use observed data

The use of data obtained by remote sensing is an alternative possibility to the use of station data, and in some aspects, it is better due to possible biases introduced by the location of climate this data in the modeling structure proposed in this article leads to higher computational costs due to the enormous growth in the number of observations, but which may be feasible using a computationally feasible spatial grid. We leave this possibility as a possible extension of our work. However, it is important to note that the results obtained in^113^ seem to indicate a consistency in the general climate trends estimated for the Cerrado using data from INMET Network stations, remote sensing data obtained from the MODIS system (MOD11A2) and reanalysis data from the NCEP/DOE Reanalysis II dataset, supporting our results in terms of the use of meteorological station data.

## Results

We first estimated the full model allowing for different trends, seasonal and cycle components for each of the country’s biomes, according to Eq. (1). As discussed in section “Methodology and data” we use components with mixed sample frequencies. The trend in this specification may vary weekly, whereas the seasonal and the cycle components are specified with a monthly variation due to their low-frequency nature. We also add as a fixed covariate in the model the latitude of each station, for controlling for the effects of angle of sunlight and atmospheric circulation related to latitude effects in the temperature. Latitude is measured in deviations from its average. Note that the model also includes the average temperature of each biome as a fixed effect $\alpha _b$, and thus the components of trend, seasonal, cycle and spatial effects are parameterized as the deviation from this mean.

**Figure 2:**
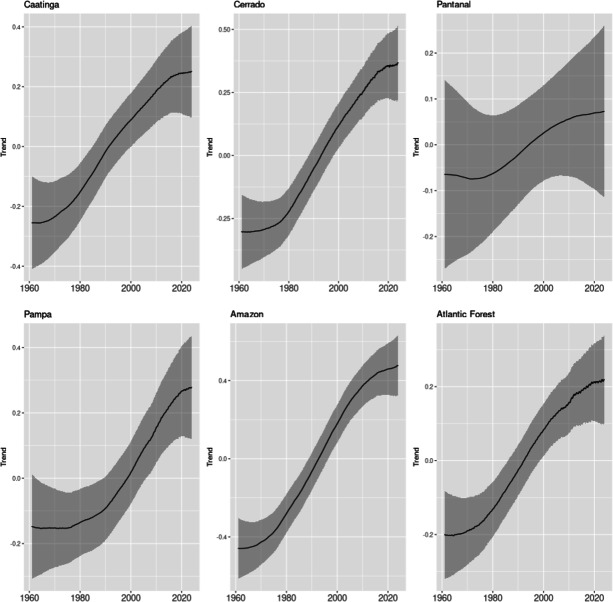
Trend components. The figure shows the posterior mean and a 95% credibility interval for the estimated trend components for each biome, for the sample period 1961–2023. Trend components are defined on a weekly frequency.

**Figure 3:**
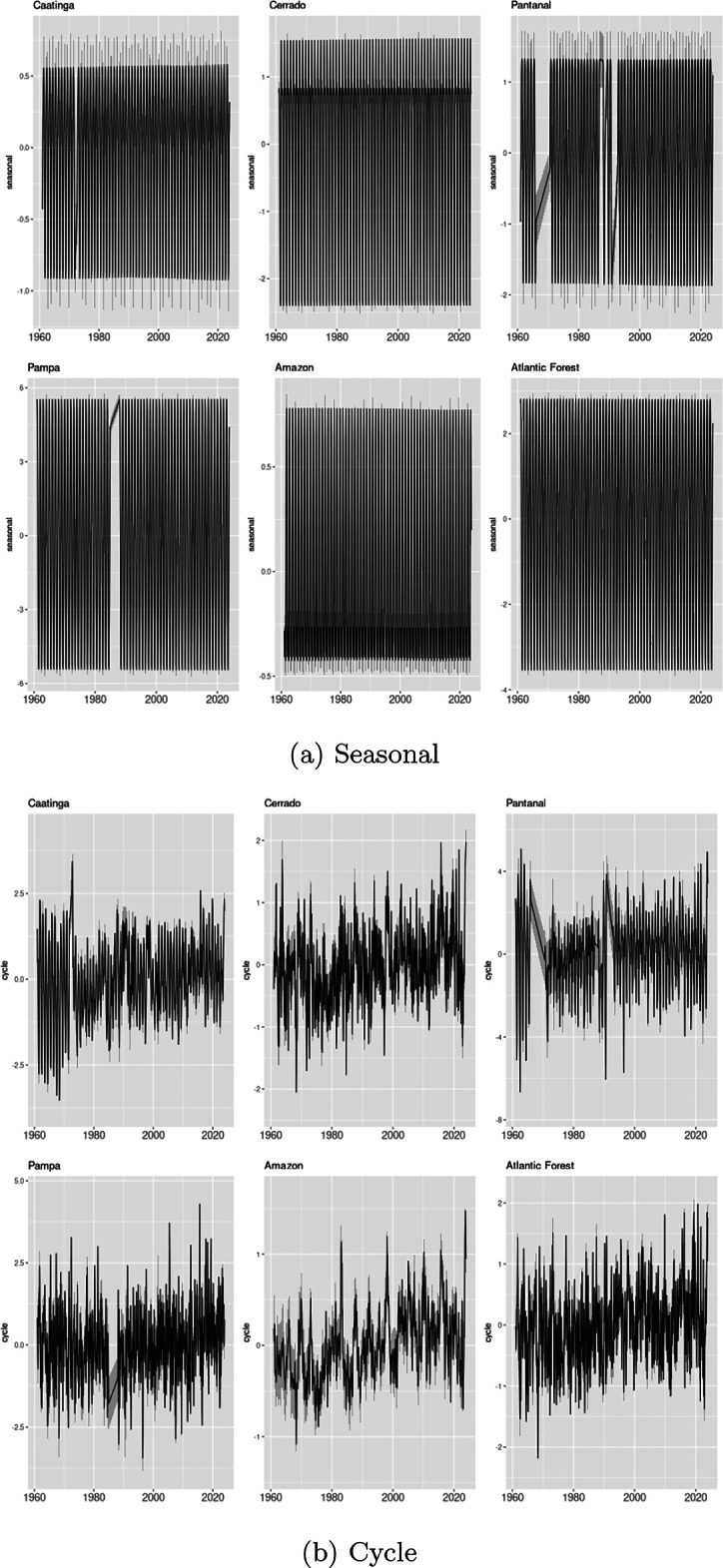
Seasonal and cycle components. The figure shows the posterior mean and a 95% credibility interval for the estimated seasonal and cycle components for each biome, for the sample period 1961–2023. Trend components are defined on a monthly frequency.

Table 1 shows the estimated parameters for the full model with biome specific trend, seasonal and cycle components, and the DIC, WAIC and the log Marginal Likelihood of the model, and Figs. 2 and 3 show the posterior mean and the 95% credibility interval for the Trend, and Seasonal and Cycles components for each biome. The estimated Trend components in Fig. 2 are summed with the average means $\alpha _b$ of each biome.

An important result it’s the growth pattern observed in the trend component for all biomes. The model captured an increase of 1.1211 °C in the trend for the Amazon biome between 1961 and 2023, followed by an increase of 0.8501 °C in the Cerrado, 0.6155 °C in Atlantic Forest, 0.5018 °C in Caatinga, 0.3782 °C in Pampa and 0.1743 °C in the Pantanal biome. The results seem to indicate that the estimated heterogeneous trends indicate a composite of global warming effects with the effects of deforestation on temperature, a result consistent with other studies on the impact of deforestation on climate change.

The observed significant temperature increases within various biomes align with previous research suggesting a complex interplay between global warming and local deforestation effects. Studies focusing on the Amazon biome^81–83^ have underscored the correlation between deforestation activities and temperature rises. Furthermore, these temperature fluctuations have been shown to exacerbate rainfall variability and contribute to heightened fire occurrences^34,85^. Similar findings have been documented for other key Brazilian biomes. For instance, investigations into the Cerrado biome have highlighted analogous trends^86^. Likewise, studies on the Atlantic Forest^87^, Caatinga^88^, and Pampa^89^ have reported temperature increases associated with deforestation activities and their ecological repercussions.

Additionally, climate change-induced alterations in extreme drought patterns have been observed in the Pantanal, with evidence suggesting a linkage to climate variations in the Amazon^72,90^.

In the seasonal component there are also great differences between biomes. While the Pampas presents a range close to 10 °C between seasons (^39^), the Amazon presents a range of about 1.25 °C (^40^), which is consistent with the relatively constant temperature of the Amazon throughout the year and the greater intra-annual variability of the Pampas, with colder winters compared to the rest of the country. The estimated model cannot identify relevant changes in seasonal patterns in the biomes studied, but this may be linked to the relatively smaller sample available to analyze these patterns. Note that we only have 62 years available to estimate the seasonal pattern, given that this is fact an annual pattern. The seasonal components also show the effect of missing data on the estimation of this component. The seasonal pattern is not accurately estimated for periods with missing data in the Pantanal and Pampa.

**Figure 4:**
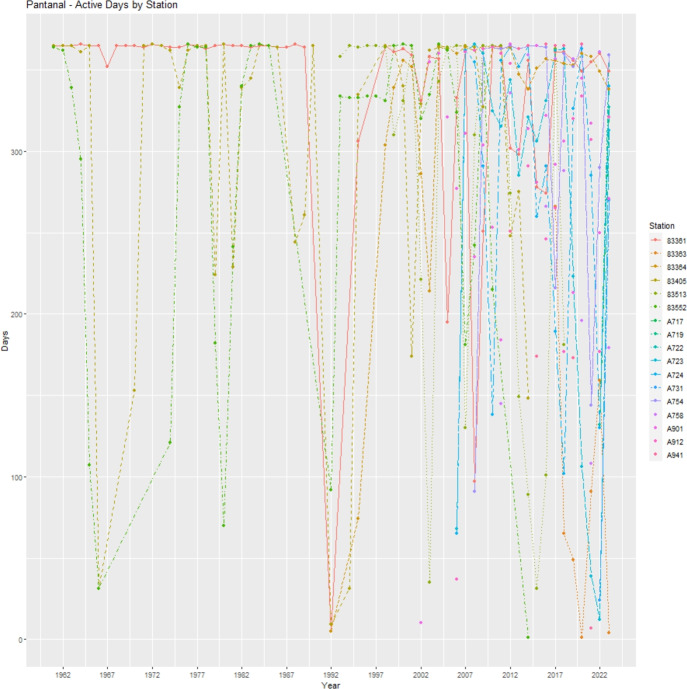
Active days by station–Pantanal. The figure shows the number of active days for each Station in Pantanal Biome.

To illustrate the problem of missing data in some sample periods, we show in Fig. 4 the number of active days for each station in the Pantanal biome. Stations with numerical codes are manually operated stations, and stations starting with A are automatic stations, which began to be operated from 2002 onwards. The Pantanal was monitored until the 2000s with only six manual stations, and in relevant periods between 1965 and 1974 and between 1991 and 1993, with fewer stations, especially in the period between 1991 and 1993 with even fewer operating stations. This pattern of missing data significantly affects the estimation of the seasonality and cycle components in these periods for the Pantanal biome. A similar problem occurs for the Pampa biome in the period from 1991 to 1993.

As we explained in section “Methodology and data”, to identify a cyclical component, the estimated AR(2) process must have complex roots. Converting the PACF(2) for AR(2) parameters we found complex roots for the Caatinga, Pantanal and Pampas, with periods of 11.1229, 9.4606 and 12.1924 months, respectively. For the other biomes, the cycle must be interpreted as a second-order autoregressive process without complex roots.

**Table 1:** Estimated parameters–model with biome specific trend, seasonal and cycle components.

	Mean	SD	0.025 q	0.5 q	0.975 q	Mode
Latitude	− 0.120	0.572	− 1.241	− 0.120	1.001	− 0.120
Precision $\epsilon \left( s,t\right)$	0.358	0.001	0.357	0.358	0.359	0.358
Avg. Temp. $\alpha$ Caatinga	25.626	0.001	25.625	25.626	25.628	25.626
Avg. Temp. $\alpha$ Cerrado	23.904	0.001	23.903	23.904	23.906	23.904
Avg. Temp. $\alpha$ Pantanal	25.672	0.001	25.671	25.672	25.673	25.672
Avg. Temp. $\alpha$ Pampa	18.756	0.001	18.755	18.756	18.757	18.756
Avg. Temp. $\alpha$ Amazon	26.548	0.001	26.547	26.548	26.549	26.548
Avg. Temp. $\alpha$ Atlantic For.	20.961	0.000	20.961	20.961	20.962	20.961
Precision $\eta _{\mu }$ trend Caatinga	119881.770	3634.102	112886.368	119826.726	127193.783	119717.007
Precision $\eta _{\mu }$ trend Cerrado	75539.991	2302.625	71108.153	75504.921	80173.549	75435.016
Precision $\eta _{\mu }$ trend Pantanal	103457.907	3134.551	97424.034	103410.455	109764.724	103315.869
Precision $\eta _{\mu }$ trend Pampa	104324.032	3163.640	98234.290	104276.096	110689.498	104180.546
Precision $\eta _{\mu }$ trend Amazon	69209.869	2088.378	65189.461	69178.383	73411.386	69115.621
Precision $\eta _{\mu }$ trend Atlantic For.	126823.702	3865.261	119384.250	126764.841	134601.717	126647.516
Precision $\eta _{s}$ seasonal Caatinga	16372.791	496.866	15416.382	16365.257	17372.536	16350.240
Precision $\eta _{s}$ seasonal Cerrado	81440.006	2468.917	76687.514	81402.609	86407.608	81328.064
Precision $\eta _{s}$ seasonal Pantanal	19278.862	585.318	18152.204	19269.982	20456.592	19252.284
Precision $\eta _{s}$ seasonal Pampa	19456.641	590.600	18319.811	19447.683	20644.994	19429.828
Precision $\eta _{s}$ seasonal Amazon	82077.244	2487.184	77289.543	82039.585	87081.554	81964.521
Precision $\eta _{s}$ seasonal Atlantic For.	22669.151	688.086	21344.671	22658.716	24053.658	22637.915
Precision $\eta _{c}$ cycle Caatinga	0.836	0.023	0.792	0.835	0.882	0.835
PACF1 cycle Caatinga	0.815	0.004	0.806	0.815	0.823	0.815
PACF2 cycle Caatinga	− 0.584	0.009	− 0.602	− 0.584	− 0.565	− 0.584
Precision $\eta _{c}$ cycle Cerrado	2.638	0.071	2.502	2.637	2.780	2.635
PACF1 cycle Cerrado	0.496	0.011	0.474	0.496	0.517	0.496
PACF2 cycle Cerrado	0.130	0.014	0.102	0.130	0.157	0.130
Precision $\eta _{c}$ cycle Pantanal	0.331	0.009	0.313	0.330	0.349	0.330
PACF1 cycle Pantanal	0.717	0.007	0.704	0.717	0.730	0.717
PACF2 Cycle Pantanal	− 0.415	0.012	− 0.439	− 0.415	− 0.392	− 0.415
Precision for $\eta _{c}$ Pampa	0.793	0.021	0.752	0.792	0.835	0.792
PACF1 cycle Pampa	0.247	0.013	0.221	0.247	0.273	0.247
PACF2 cycle Pampa	− 0.021	0.014	− 0.049	− 0.021	0.007	− 0.021
Precision $\eta _{c}$ cycle Amazon	8.858	0.247	8.381	8.855	9.355	8.848
PACF1 cycle Amazon	0.727	0.007	0.713	0.727	0.740	0.727
PACF2 cycle Amazon	0.087	0.014	0.059	0.087	0.116	0.087
Precision $\eta _{c}$ cycle Atlantic For.	2.348	0.063	2.227	2.347	2.473	2.345
PACF1 cycle Atlantic For.	0.397	0.012	0.373	0.397	0.420	0.397
PACF2 cycle Atlantic For.	0.096	0.014	0.069	0.096	0.124	0.096
$\log \tau$ Matérn	− 3.331	0.024	− 3.377	− 3.331	− 3.284	− 3.331
$\log \kappa$ Matérn	− 1.104	0.031	− 1.164	− 1.104	− 1.043	− 1.104
DIC	3760738.41					
WAIC	3760399.15					
Marginal log-likelihood	− 1887108.57					

The Table summarizes the estimated parameters of the model with specific components of trend, seasonality and cycle for each biome, showing the mean, standard deviation, the quantiles of 0.025, 0.5, 0.975 and the mode of the posterior distribution of each parameter. The parameters were estimated using Bayesian inference through integrated nested Laplace approximations. See Online Methods for details on the interpretation of each parameter.

**Figure 5:**
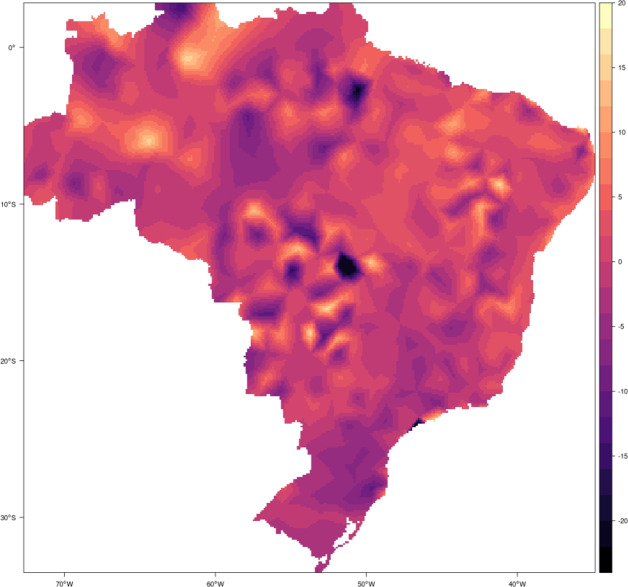
Spatial random effects. The figure shows the posterior average of the estimated spatial random effects. These effects are obtained as a spatially continuous projection of the Matérn spatial covariance function.

The estimated spatial random effects are shown in Fig. 5. As discussed in^24^, spatial random effects are a way of capturing all systematic patterns not captured by the other components in the model that present spatial dependence, and in this aspect, they serve to incorporate the effects of altitude, local weather patterns and other effects that generate spatial dependence. The high temperature range of spatial effects is also consistent with the climatic variability observed in Brazil and it’s large and geographically diverse territory (e.g.,^104^).

The model with individual components for biome presented in Table 1 indicates relevant evidence for the existence of heterogeneity in the permanent effects of climate change on temperature patterns for Brazilian biomes, but to proceed to a statistical test for the validity of this evidence we compare the results of this model with those obtained by a restricted specification, assuming a single global trend for all biomes, using the results of the Bayes Factor, and DIC and WAIC criteria, as explained in section 2. The estimate for the restricted model is shown in Table 2. Note also that in the restricted model we do not assume unique components of seasonality and cycles in the restricted model, as these components must vary in relation to the biome, as is evident from the patterns observed in Fig. 3. Our objective is to verify the hypothesis of heterogeneity in the permanent component of temperature change, measured by the trend.

**Table 2:** Estimated parameters–restricted model with a global trend component.

	Mean	Sd	0.025 quant	0.5 quant	0.975 quant	Mode
Latitude	− 0.247	0.936	− 2.081	− 0.247	1.587	− 0.247
Precision $\epsilon \left( s,t\right)$	0.357	0.001	0.356	0.357	0.358	0.357
Avg. Temp. $\alpha$ Caatinga	25.635	0.000	25.635	25.635	25.636	25.635
Avg. Temp. $\alpha$ Cerrado	23.918	0.000	23.918	23.918	23.919	23.918
Avg. Temp. $\alpha$ Pantanal	25.672	0.000	25.672	25.672	25.673	25.672
Avg. Temp. $\alpha$ Pampa	18.756	0.000	18.755	18.756	18.756	18.756
Avg. Temp. $\alpha$ Amazon	26.551	0.000	26.551	26.551	26.551	26.551
Avg. Temp. $\alpha$ Atlantic For.	20.959	0.000	20.959	20.959	20.959	20.959
Precision $\eta _{\mu }$ trend	1205005.245	12077.035	1181403.681	1204944.730	1228954.865	1204824.081
Precision $\eta _{s}$ seasonal Caatinga	20190.351	202.779	19794.075	20189.332	20592.483	20187.302
Precision $\eta _{s}$ Seasonal Cerrado	83955.876	843.050	82308.367	83951.644	85627.729	83943.206
Precision $\eta _{s}$ Seasonal Pantanal	21053.425	211.443	20640.219	21052.363	21472.737	21050.247
Precision $\eta _{s}$ seasonal Pampa	20352.568	204.401	19953.123	20351.542	20757.916	20349.495
Precision $\eta _{s}$ seasonal Amazon	84572.164	849.211	82912.615	84567.900	86256.232	84559.401
Precision $\eta _{s}$ seasonal Atlantic For.	21013.547	211.037	20601.133	21012.487	21432.055	21010.375
Precision $\eta _{c}$ cycle Caatinga	1.048	0.010	1.028	1.048	1.068	1.048
PACF1 cycle Caatinga	0.812	0.002	0.809	0.812	0.815	0.812
PACF2 cycle Caatinga	− 0.424	0.004	− 0.432	− 0.424	− 0.416	− 0.424
Precision $\eta _{c}$ cycle Cerrado	1.597	0.016	1.566	1.597	1.628	1.596
PACF1 cycle Cerrado	0.663	0.003	0.657	0.663	0.668	0.663
PACF2 cycle Cerrado	0.096	0.005	0.086	0.096	0.106	0.096
Precision $\eta _{c}$ cycle Pantanal	0.315	0.003	0.309	0.315	0.322	0.315
PACF1 cycle Pantanal	0.655	0.003	0.649	0.655	0.661	0.655
PACF2 cycle Pantanal	− 0.344	0.004	− 0.353	− 0.344	− 0.336	− 0.344
Precision $\eta _{c}$ cycle Pampa	0.445	0.004	0.436	0.445	0.453	0.445
PACF1 cycle Pampa	0.141	0.005	0.132	0.141	0.151	0.141
PACF2 cycle Pampa	0.101	0.005	0.091	0.101	0.110	0.101
Precision $\eta _{c}$ cycle Amazon	5.100	0.051	5.001	5.100	5.201	5.099
PACF1 cycle Amazon	0.884	0.001	0.882	0.884	0.887	0.884
PACF2 cycle Amazon	− 0.201	0.005	− 0.210	− 0.201	− 0.192	− 0.201
Precision $\eta _{c}$ cycle Atlantic For.	1.099	0.011	1.078	1.099	1.121	1.099
PACF1 cycle Atlantic For.	0.401	0.004	0.392	0.401	0.409	0.401
PACF2 cycle Atlantic For.	0.237	0.005	0.228	0.237	0.246	0.237
$\log \tau$ Matérn	− 3.121	0.010	− 3.140	− 3.121	− 3.102	− 3.121
$\log \kappa$ Matérn	− 1.607	0.010	− 1.627	− 1.607	− 1.588	− 1.607
DIC	3761260.49					
WAIC	3760978.41					
Marginal log-likelihood	− 1887600.81					

The Table summarizes the estimated parameters of the model with a global trend, and specific seasonality and cycle for each biome, showing the mean, standard deviation, the quantiles of 0.025, 0.5, 0.975 and the mode of the posterior distribution of each parameter. The parameters were estimated using Bayesian inference through integrated nested Laplace approximations. See Online Methods for details on the interpretation of each parameter.

To obtain the Bayes factor we compute the difference between the estimated log Marginal Likelihood of unrestricted (Table 1) and restricted models (Table 2). The log difference is calculated as 492.2425, indicating a relevant difference according to Jeffreys’ criteria^100^ for the Bayes factor, and thus indicating severe posterior evidence in favor of the unrestricted model, indicating strong statistical evidence in favor of the model with spatial heterogeneity in temperature trends between the biomes of Brazil. The DIC and WAIC information criteria also supports the better support for the unrestricted model, with a DIC of 3760738 for the unrestricted trend model versus a DIC of 3761260.49 for the global trend model, and similarly a WAIC of 3760399.15 for the unrestricted model and 3760978.41 for the restricted model, remembering that in comparative analyzes of information criteria, a lower value is indicative of a better fit/complexity relationship between the compared models.

## Conclusion

We present a methodology to test the spatial heterogeneity of climate change, testing the presence of multiple trends in warming patterns for Brazilian Biomes through a spatio-temporal statistical model, decomposing the observed variations into permanent and transitory components. The results of the proposed specification test and specification analyzes using information criteria indicate strong statistical support for the presence of multiple trends and rejection of the hypothesis of a single global trend.

The results found are especially relevant as they indicate a trend of more accelerated growth in the increase in temperature for the Amazon and Cerrado biomes and to a lesser extent for the Atlantic Forest. These results are consistent with other work showing the positive relationship between deforestation and increased land temperature^47,82,114–116^.

The largest temperature increases observed for these biomes also support other works indicating the composition of global warming effects with local deforestation effects, as the relationship between deforestation and temperature increases found for the Amazon biome^81–84^ and the related impacts on rainfall variability and fires in this biome^34,85^. Comparable results were also found for the Cerrado^86^, Atlantic Forest^87^, Caatinga^88^, Pampa^89^, and changes in extreme drought patterns in the Pantanal due to climate change in the Amazon^72,90^.

The proposed statistical framework also permits to directly verify the spatial heterogeneity in other climate change effects, as precipitation patterns and changes in the timing of biological events (phenology), and thus is a useful tool for statistical analysis of climate change patterns.

The method used involves some important simplifications. The first relevant simplification is to use the latent component of spatial random effect as a way to capture the effect of all possible covariates that affect the spatial distribution of temperature, and also that this spatial effect is time invariant, as discussed in^24^. In this way, this spatial component captures the composite effect of variables such as altitude, distance to the sea and other climatic effects that are spatially localized. We tested alternative specifications where the spatial effect is made time-varying, but the overall results indicate that for the estimated sample period the assumption of spatial random effects appears valid, which can be explained by the limited sample period used in the analysis. As these effects should not vary significantly over the period of time studied, this assumption seems appropriate in the analysis carried out.

Another limitation is assuming a structure of separability between the trend, seasonality and cycle components and the spatial effects. This structure is imposed on the model and allows the statistical identification of latent parameters and effects. This assumption could be violated if there was a possible interaction between the components and the spatial effect, such as spatial heterogeneity in the trend. Note that by allowing specific trends, seasonality and cycles for each biome we are controlling this possibility, but it is still possible that there are variations in these components within each biome analyzed. One possibility to be explored in future work is to estimate the model using a greater disaggregation in the observed biomes, which would be important for biomes with greater climate variability or spatial extension.

Our analyzes suffer from the same problem of the time frame limited by available observations of temperature data affecting estimates of climate change patterns, especially for such a wide spatial region. The precision of the estimates, especially the seasonality and cycle components, is greatly affected by the number of observations over time, since we are estimating periodic patterns of variation, and thus the precision of these components depends on the number of repetitions of the same patterns. This limitation makes it especially difficult to estimate relevant variations in the pattern of seasonal variation, as discussed in^35^. As our main discussion is based on the trend component, we are less exposed to the problem of sample size, since this component is estimated using a weekly frequency, which allows greater precision for this estimation, allowing us to estimate the variations observed in the trend more accurately.

In our analysis we only model climate change patterns in temperatures, but an extension of the work is to model the joint dynamics of temperature with other relevant climate measures, such as rainfall and relative humidity. In this joint modeling it is possible to analyze the joint dynamics and interactions between these measures, allowing us to verify how the relationships between variables are altered due to global and local patterns of climate change. Examples of joint modeling of climate variables can be seen in^117^ and^118^. In this aspect, joint modeling can be seen as a spatio-temporal generalization of joint models for climate variation measurements.

A further use of the method is its application to maximum temperature series, particularly in the analysis of temperature records, as examined in.^35^. This work analyzes patterns of climate change in annual temperature and daily rainfall records, as well as modeling the probability of occurrence of rain and the duration of droughts for a single meteorological station. Therefore, a possible extension to the present work is to carry out these analyzes of temperature and rainfall records and other climate measures for the set of climate measures for the different biomes analyzed.

## Data Availability

The datasets analysed during the current study are available in the INMET website: https://bdmep.inmet.gov.br/.

## Appendix 1

### Spatial covariance function

The ${\mathcal {C}}\left( h\right)$ is a covariance function of the Matérn class given by:

2 $$\begin{aligned} {\mathcal {C}}\left( h\right) =\frac{1}{\Gamma (\nu )2^{\nu -1}}\left( kh\right) ^{\nu }K_{\nu }\left( kh\right) \end{aligned}$$

with $K_{\nu }$ being a modified Bessel function of the second type. We use the following representation in terms of $\log \tau$ and $\log \kappa$ for the covariance function:

$$\begin{aligned} \log \tau =\frac{1}{2}\log \left( \frac{\Gamma (\nu )}{\Gamma (\alpha )(4\pi )^{d/2}}\right) -\log \sigma -\nu \log \rho \end{aligned}$$

$$\begin{aligned} \log \kappa =\frac{\log (8\nu )}{2}-\log \rho \end{aligned}$$

The primary benefit of this format is that, given the value of $\nu$, it reduces the number of parameters needing estimation to just two.

## Code availability

Custom code developed by the authors, using r-inla (www.r-inla.org) library.

## References

[1] Ellery M, Scholes W, Mentis RJ (1991). An initial approach to predicting the sensitivity of the South African grassland biome to climate change. S. Afr. J. Sci..

[2] Hansen AJ (2001). Global change in forests: Responses of species, communities, and biomes: interactions between climate change and land use are projected to cause large shifts in biodiversity. Bioscience.

[3] Salazar LF, Nobre CA, Oyama MD (2007). Climate change consequences on the biome distribution in tropical South America. Geophys. Res. Lett..

[4] Salazar LF, Nobre CA (2010). Climate change and thresholds of biome shifts in Amazonia. Geophys. Res. Lett..

[5] de Oliveira G, Araújo M B, Rangel T F (2012). Conserving the Brazilian semiarid (Caatinga) biome under climate change. Biodivers. Conserv..

[6] Grimm NB (2013). The impacts of climate change on ecosystem structure and function. Front. Ecol. Environ..

[7] Huntley B (2021). Projected climatic changes lead to biome changes in areas of previously constant biome. J. Biogeogr..

[8] Boonman CC (2022). Trait-based projections of climate change effects on global biome distributions. Divers. Distrib..

[9] Giglio S, Kelly B, Stroebel J (2021). Climate finance. Annu. Rev. Financ. Econ..

[10] Burrell AL, Evans JP, De Kauwe MG (2020). Anthropogenic climate change has driven over 5 million km^2^ of drylands towards desertification. Nat. Commun..

[11] Jain P, Castellanos-Acuna D, Coogan S C P (2022). Observed increases in extreme fire weather driven by atmospheric humidity and temperature. Nat. Clim. Chang..

[12] Swati S (2022). Forest fire emissions: A contribution to global climate change. Front. For. Global Change.

[13] Brown P (2023). Climate warming increases extreme daily wildfire growth risk in California. Nature.

[14] Gibson DJ, Newman JA (2019). Grasslands and Climate Change.

[15] Chang J, Ciais P, Gasser T (2021). Climate warming from managed grasslands cancels the cooling effect of carbon sinks in sparsely grazed and natural grasslands. Nat. Commun..

[16] Wu GL, Cheng Z, Alatalo JM, Zhao J, Liu Y (2021). Climate warming consistently reduces grassland ecosystem productivity. Earth’s Futur..

[17] Carozzi M, Martin R, Klumpp K, Massad RS (2022). Effects of climate change in European croplands and grasslands: Productivity, greenhouse gas balance and soil carbon storage. Biogeosciences.

[18] Priya A (2023). Impact of climate change and anthropogenic activities on aquatic ecosystem–a review. Environ. Res..

[19] Woolway RI, Sharma S, Smol JP (2022). Lakes in hot water: The impacts of a changing climate on aquatic ecosystems. Bioscience.

[20] Magnan AK, Pörtner H-O, Duvat VKE (2021). Estimating the global risk of anthropogenic climate change. Nat. Clim. Chang..

[21] Abbass K (2022). A review of the global climate change impacts, adaptation, and sustainable mitigation measures. Environ. Sci. Pollut. Res..

[22] Guan Y (2021). Changes in global climate heterogeneity under the 21st century global warming. Ecol. Ind..

[23] Kaufmann RK (2017). Spatial heterogeneity of climate change as an experiential basis for skepticism. Proc. Natl. Acad. Sci..

[24] Laurini MP (2019). A spatio-temporal approach to estimate patterns of climate change. Environmetrics.

[25] Bloomfield P (1992). Trends in global temperature. Clim. Change.

[26] Barasal Morales, A., Laurini, M. & Vrieling, A. Climate risk premium: Assessing the influence of global warming effects on stock market dynamics. *SSRN Electron. J.* (2021). Available at SSRN: https://ssrn.com/abstract=4614201 .

[27] Clark PK (1987). The cyclical component of US economic activity. Q. J. Econ..

[28] Cressie NAC, Wikle CK (2011). Statistics for Spatio-Temporal Data.

[29] Harvey AC (1990). Forecasting, Structural Time Series Models and the Kalman Filter.

[30] Lindgren F, Rue H, Lindström J (2011). An explicit link between Gaussian fields and Gaussian Markov random fields: The stochastic partial differential equation approach. J. R. Stat. Soc. Ser. B Stat Methodol..

[31] Valente F, Laurini M (2020). Tornado occurrences in the United States: A spatio-temporal point process approach. Econometrics.

[32] Valente F, Laurini M (2021). Pre-harvest sugarcane burning: A statistical analysis of the environmental impacts of a regulatory change in the energy sector. Clean. Eng. Technol..

[33] Valente F, Laurini M (2021). Spatio-temporal analysis of fire occurrence in Australia. Stoch. Env. Res. Risk Assess..

[34] Valente F, Laurini M (2023). A spatio-temporal analysis of fire occurrence patterns in the Brazilian Amazon. Sci. Rep..

[35] Valente F, Laurini M (2022). Urban climate change: A statistical analysis for São Paulo. Urban Clim..

[36] da Silva JMC, Lacher TE, Goldstein MI, DellaSala DA (2020). Caatinga-South America. Encyclopedia of the World’s Biomes.

[37] Colli GR, Vieira CR, Dianese JC (2020). Biodiversity and conservation of the Cerrado: Recent advances and old challenges. Biodivers. Conserv..

[38] Wantzen KM (2024). The end of an entire biome? world’s largest wetland, the Pantanal, is menaced by the Hidrovia project which is uncertain to sustainably support large-scale navigation. Sci. Total Environ..

[39] Roesch LFW (2009). The Brazilian Pampa: A fragile biome. Diversity.

[40] Flores BM, Montoya E, Sakschewski B (2024). Critical transitions in the Amazon forest system. Nature.

[41] Colombo A, Joly C (2010). Brazilian Atlantic Forest lato sensu: The most ancient Brazilian forest, and a biodiversity hotspot, is highly threatened by climate change. Braz. J. Biol..

[42] Heinrich V H A, Dalagnol R, Cassol H L G (2021). Large carbon sink potential of secondary forests in the Brazilian Amazon to mitigate climate change. Nat. Commun..

[43] Feng Y, Zeng Z, Searchinger T D (2022). Doubling of annual forest carbon loss over the tropics during the early twenty-first century. Nat. Sustain..

[44] Zou Y (2020). Using CESM-RESFire to understand climate-fire-ecosystem interactions and the implications for decadal climate variability. Atmos. Chem. Phys..

[45] Valente F, Laurini M (2024). The dynamics of fire activity in the Brazilian Pantanal: A log-Gaussian cox process-based structural decomposition. Fire.

[46] Lopes dos Santos G (2021). Degradation of the Brazilian Cerrado: Interactions with human disturbance and environmental variables. For. Ecol. Manage..

[47] Scott CE, Monks SA, Spracklen DV (2018). Impact on short-lived climate forcers increases projected warming due to deforestation. Nat. Commun..

[48] Flannigan M, Stocks B, Wotton B (2000). Climate change and forest fires. Sci. Total Environ..

[49] Heidari H, Warziniack T, Brown TC, Arabi M (2021). Impacts of climate change on hydroclimatic conditions of U.S. National forests and grasslands. Forests.

[50] Jasechko S, Sharp Z, Gibson J (2013). Terrestrial water fluxes dominated by transpiration. Nature.

[51] Smith C, Baker JCA, Spracklen DV (2023). Tropical deforestation causes large reductions in observed precipitation. Nature.

[52] Leite-Filho AT, Soares-Filho BS, Davis JL (2021). Deforestation reduces rainfall and agricultural revenues in the Brazilian Amazon. Nat. Commun..

[53] Chagas VBP, Chaffe PLB, Blöschl G (2022). Climate and land management accelerate the Brazilian water cycle. Nat. Commun..

[54] Xu X (2022). Deforestation triggering irreversible transition in Amazon hydrological cycle. Environ. Res. Lett..

[55] Latrubesse EM (2019). Fostering water resource governance and conservation in the Brazilian Cerrado biome. Conserv. Sci. Pract..

[56] Althoff D, Rodrigues LN, da Silva DD (2021). Assessment of water availability vulnerability in the Cerrado. Appl. Water Sci..

[57] Salmona YB (2023). A worrying future for river flows in the Brazilian Cerrado provoked by land use and climate changes. Sustainability.

[58] Mu Y, Jones C (2022). An observational analysis of precipitation and deforestation age in the Brazilian legal Amazon. Atmos. Res..

[59] Lawrence D, Vandecar K (2015). Effects of tropical deforestation on climate and agriculture. Nat. Clim. Chang..

[60] Werth D, Avissar R (2002). The local and global effects of amazon deforestation. J. Geophys. Res. Atmos..

[61] Gomes WB, Correia FWS, Capistrano VB, Veiga JAP (2020). Water budget changes in the Amazon basin under RCP 8.5 and deforestation scenarios. Clim. Res..

[62] Hofmann G S, Silva R C, Weber E J (2023). Changes in atmospheric circulation and evapotranspiration are reducing rainfall in the Brazilian Cerrado. Sci. Rep..

[63] Bottino MJ, Nobre P, Giarolla E (2024). Amazon savannization and climate change are projected to increase dry season length and temperature extremes over Brazil. Sci. Rep..

[64] Anjos LJS, de Toledo PM (2018). Measuring resilience and assessing vulnerability of terrestrial ecosystems to climate change in South America. PLoS ONE.

[65] de Souza Batista F, Duku C, Hein L (2023). Deforestation-induced changes in rainfall decrease soybean-maize yields in Brazil. Ecol. Model..

[66] Rodrigues AA (2022). Cerrado deforestation threatens regional climate and water availability for agriculture and ecosystems. Global Change Biol..

[67] Filho WLFC, de Oliveira-Júnior JF, Santiago DB (2023). The assessment of climatic, environmental, and socioeconomic aspects of the Brazilian Cerrado. Ecol. Process..

[68] de Paula Sousa Júnior V, Sparacino J, de Espindola G M, Sousa de Assis R J (2022). Land-use and land-cover dynamics in the Brazilian Caatinga dry tropical forest. Conservation.

[69] Araujo HFP, Canassa NF, Machado CCC (2023). Human disturbance is the major driver of vegetation changes in the Caatinga dry forest region. Sci. Rep..

[70] Webb TJ, Woodward FI, Hannah L, Gaston KJ (2005). Forest cover-rainfall relationships in a biodiversity hotspot: The Atlantic forest of Brazil. Ecol. Appl..

[71] Lázaro WL, Oliveira-Júnior ES (2020). Climate change reflected in one of the largest wetlands in the world: an overview of the Northern Pantanal water regime. Acta Limnol. Bras..

[72] Marengo JA (2021). Extreme drought in the Brazilian Pantanal in 2019–2020: Characterization, causes, and impacts. Front. Water.

[73] Hirota M, Holmgren M, Nes EHV, Scheffer M (2011). Global resilience of tropical forest and savanna to critical transitions. Science.

[74] Ouyang Z, Sciusco P, Jiao T (2022). Albedo changes caused by future urbanization contribute to global warming. Nat. Commun..

[75] Hasler N, Williams CA, Denney VC (2024). Accounting for albedo change to identify climate-positive tree cover restoration. Nat. Commun..

[76] Anjos L J, Barreiros de Souza E, Amaral C T, Igawa T K,  & Mann de Toledo P (2021). Future projections for terrestrial biomes indicate widespread warming and moisture reduction in forests up to 2100 in South America. Global Ecol. Conserv..

[77] Anadón JD, Sala OE, Maestre FT (2014). Climate change will increase savannas at the expense of forests and treeless vegetation in tropical and subtropical Americas. J. Ecol..

[78] Zelazowski P, Malhi Y, Huntingford C, Sitch S, Fisher JB (2011). Changes in the potential distribution of humid tropical forests on a warmer planet. Philos. Trans. Royal Soc. A Math. Phys. Eng. Sci..

[79] Boit A (2016). Large-scale impact of climate change vs. Land-use change on future biome shifts in Latin America. Glob. Change Biol..

[80] Pinho P F, Anjos L J S, Rodrigues-Filho S, Santos D V, Toledo P M (2020). Projections of Brazilian biomes resilience and socio-environmental risks to climate change. Sustain. Debate.

[81] Alves L, Marengo J, Fu R, Bombardi R (2017). Sensitivity of Amazon regional climate to deforestation. Am. J. Clim. Chang..

[82] AlvesdeOliveira BF, Bottino MJ, Nobre CP (2021). Deforestation and climate change are projected to increase heat stress risk in the Brazilian Amazon. Commun. Earth Environ..

[83] Gatti L V, Basso L S, Miller J B (2021). Amazonia as a carbon source linked to deforestation and climate change. Nature.

[84] Butt EW (2023). Amazon deforestation causes strong regional warming. Proc. Natl. Acad. Sci..

[85] da Silva RM, Lopes AG, Santos CAG (2023). Deforestation and fires in the Brazilian Amazon from 2001 to 2020: Impacts on rainfall variability and land surface temperature. J. Environ. Manage..

[86] Rodrigues AA (2022). Cerrado deforestation threatens regional climate and water availability for agriculture and ecosystems. Glob. Change Biol..

[87] Wanderley LNR, Domingues M, Joly A, da Rocha R (2019). Relationship between land surface temperature and fraction of anthropized area in the Atlantic forest region, Brazil. PLoS ONE.

[88] Araujo HFP, Canassa NF, Machado CCC, Tabarelli M (2023). Human disturbance is the major driver of vegetation changes in the Caatinga dry forest region. Sci. Rep..

[89] Prevedello JA, Winck GR, Weber MM, Nichols E, Sinervo B (2019). Impacts of forestation and deforestation on local temperature across the globe. PLoS ONE.

[90] Bergier I (2018). Amazon rainforest modulation of water security in the Pantanal wetland. Sci. Total Environ..

[91] Weiskopf SR (2020). Climate change effects on biodiversity, ecosystems, ecosystem services, and natural resource management in the United States. Sci. Total Environ..

[92] Saladin B, Pellissier L, Graham CH (2020). Rapid climate change results in long-lasting spatial homogenization of phylogenetic diversity. Nat. Commun..

[93] Muluneh M (2021). Impact of climate change on biodiversity and food security: A global perspective–a review article. Agric. Food Secur..

[94] Rocque RJ (2021). Health effects of climate change: An overview of systematic reviews. BMJ Open.

[95] Kahn ME (2021). Long-term macroeconomic effects of climate change: A cross-country analysis. Energy Econ..

[96] Rue H, Martino S, Chopin N (2009). Approximate Bayesian inference for latent Gaussian models by using integrated nested Laplace approximations. J. R. Stat. Soc. Ser. B Stat. Methodol..

[97] Bakka H (2018). Spatial modeling with R-INLA: A review. Wiley Interdiscip. Rev. Comput. Stat..

[98] Spiegelhalter DJ, Best NG, Carlin BP, van der Linde A (2002). Bayesian measures of model complexity and fit (with discussion). J. Roy. Stat. Soc. B.

[99] Watanabe S (2013). A widely applicable Bayesian information criterion. J. Mach. Learn. Res..

[100] Jeffreys H (1961). Theory of Probability.

[101] Sampaio EV (1995). Overview of the Brazilian Caatinga.

[102] Tabarelli M, Leal I R, Scarano F R, Silva J M C d (2018). Caatinga: legado, trajetória e desafios rumo à sustentabilidade. Ciência e Cultura.

[103] de Castro Oliveira G, Francelino M R, Arruda D M, Fernandes-Filho E I, Schaefer C E G R (2019). Climate and soils at the Brazilian semiarid and the forest-Caatinga problem: New insights and implications for conservation. Environ. Res. Lett..

[104] Alvares C A, Stape J L, Sentelhas P C, Gonçalves J L d M, Sparovek G (2013). Köppen’s climate classification map for Brazil. Meteorol. Z..

[105] Nascimento DTF, Novais GT (2020). Cerrado climate: Atmospheric dynamics and features, variability and climatic typologies. Élisée-Revista De Geografia Da UEG.

[106] Correia Filho W, de Oliveira-Júnior J, Santiago D (2023). The assessment of climatic, environmental, and socioeconomic aspects of the Brazilian Cerrado. Ecol. Process..

[107] Schmidt IB, Eloy L (2020). Fire regime in the Brazilian Savanna: Recent changes, policy and management. Flora.

[108] Colman CB, Oliveira PTS, Almagro A, Soares-Filho BS, Rodrigues DBB (2019). Effects of climate and land-cover changes on soil erosion in Brazilian Pantanal. Sustainability.

[109] Hilty, J. A. *Climate and Conservation: Landscape and Seascape Science, Planning, and Action* (Island Press, 2012). Retrieved 6 March 2017.

[110] Marengo JA (2018). Changes in climate and land use over the amazon region: Current and future variability and trends. Front. Earth Sci..

[111] Wagner F (2020). Mapping Atlantic rainforest degradation and regeneration history with indicator species using convolutional network. PLoS ONE.

[112] Silva RFBD (2023). Toward a forest transition across the Brazilian Atlantic Forest biome. Front. For. Global Change.

[113] Hofmann GS (2021). The Brazilian Cerrado is becoming hotter and drier. Glob. Change Biol..

[114] Parsons LA (2021). Tropical deforestation accelerates local warming and loss of safe outdoor working hours. One Earth.

[115] Li Y, Brando P M, Morton D C (2022). Deforestation-induced climate change reduces carbon storage in remaining tropical forests. Nat. Commun..

[116] Lawrence D, Coe M, Walker W, Verchot L, Vandecar K (2022). The unseen effects of deforestation: Biophysical effects on climate. Front. For. Global Change.

[117] Fichera A, King R, Kath J (2023). Spatial modelling of agro-ecologically significant grassland species using the INLA-SPDE approach. Sci. Rep..

[118] Pan, J., He, K., Wang, K., Mu, Q. & Ling, C. Spatio-temporal joint analysis of pm2.5 and ozone in California with INLA (2024). arXiv:2404.14446. 10.1016/j.jenvman.2024.121294 38880600

